# Supplementary material for: Igneous meteorites suggest Aluminium-26 heterogeneity in the early Solar Nebula
Source: Nat Commun. 2023 Aug 29;14:4940. doi: 10.1038/s41467-023-40026-1 (PMC10465487; doi:10.1038/s41467-023-40026-1)
Supplement: Supplementary file 1 — Supplementary information [file 41467_2023_40026_MOESM1_ESM.pdf]

Supplementary information

Title: Igneous meteorites suggest Aluminium-26 heterogeneity in the early Solar Nebula

Krestianinov et al.

Supplementary notes

U and Pb distribution, concentration, and ratios

Batch A167 includes six fractions: two whole-rock, three pyroxene, and one plagioclase. Batch A170 includes ten smaller fractions of pyroxene and four fractions of the whole rock. Four fractions were measured at UCD: three pyroxenes and one whole-rock. For batch A167, U and Pb concentrations were measured along with Pb isotopes, while for the A170 and UCD batches only Pb isotopes were measured. U-Pb and Pb-Pb isotopic data are presented in Supplementary Data 1. Concentrations are reported relative to the weights before leaching.

Uranium and Pb distribution between minerals and whole-rock leachates is presented in Supplementary Figs. 2A and 2B, $^{206}Pb/^{204}Pb$ ratios are shown in Supplementary Fig. 2C. Uranium mainly concentrates in W2a and W2b leachates. Augitic pyroxene of brown colour has the highest U concentrations up to 320 ppb in W2b leachate, while orthopyroxene of green colour has concentrations of U in the range of 25-45 ppb in W2a, W2b and R fractions. Plagioclase has the lowest U concentrations of 8-13 ppb in W2a, W2b and R fractions. Silica oxide minerals, like tridymite and cristobalite, were not analysed in this study but usually contain a relatively low amount of U because U^{4+} is too large (103 pm [1]) to substitute for the relatively small (40 pm [1]) Si^{4+}. The whole-rock fractions show U concentration up to 85-95 ppb in W2a and W2b leachates.

Pb mainly concentrates in R fractions (Supplementary Fig. 2B). The concentrations here are from 19 ppb in plagioclase up to 94 ppb in orthopyroxene. W1a leachates of whole-rock fractions have relatively large proportions of Pb, indicating efficient removal of terrestrial Pb and/or dissolution of phosphates. The Pb concentrations here are 23-24 ppb.

$^{206}Pb/^{204}Pb$ ratios (corrected for spike, fractionation and blank) have large variations (Supplementary Fig. 2C) and indicate that early leaching steps effectively removed non-radiogenic Pb. All W1a, W1b, and W2a leachates have relatively low $^{206}Pb/^{204}Pb$ ratios of 29-156, while W2b aliquots have $^{206}Pb/^{204}Pb$ ratios in the range of 671-3580 and R aliquots up to ~ 12280 and ~ 50870 in clinopyroxene and orthopyroxene, respectively. These $^{206}Pb/^{204}Pb$ ratios are similar to ones reported for angrites [2,3].

Isochron calculation

The adequate choice of points for isochron regression is critical to obtain precise and accurate Pb-Pb age. Usually, first washes (W1a, W1b and W2a) have low ratios of radiogenic Pb to other Pb components and contain a large amount of terrestrial Pb that pulls the isochron towards the modern terrestrial Pb isotopic composition (Supplementary Fig. 3). Therefore, all first washes should be removed from the isochron calculation (Supplementary Fig. 4). Further rejection is based on consideration of the following geochemical and/or analytical criteria. First, plagioclase in most cases has a low $^{238}U/^{204}Pb$ and contains a large proportion of non-

radiogenic Pb, and therefore usually excluded from the regression. In EC 002 plagioclase is relatively radiogenic but shows open U-Pb system behaviour (Supplementary Fig. 5), so we excluded these two points. In addition, albitic plagioclase in EC 002 has lamellae of K-feldspar , which can cause incongruent dissolution of plagioclase and biased $^{207}Pb/^{206}Pb$ ratios [4]. The next step is the elimination of points with a small amount of radiogenic Pb because they are more affected by variations of Pb blank and its isotopic composition. To choose the right cut-off value, we analyse the distribution of $^{206}Pb^*$ (* denotes radiogenic) in W2b and R fractions (Supplementary Fig. 6).

Most fractions that contain less than 0.5×10^{-13} mol of radiogenic $^{206}Pb^*$ are W2b fractions from A170 analytical session. Their low content of radiogenic $^{206}Pb^*$ is explained by the small mass of these fractions and the resistance of orthopyroxene to hot 6M HCl acid. The exclusion of these 11 points yields the isochron with the age of 4565.49 ± 0.18 Ma, MSWD = 2.8 (Supplementary Fig. 7). A regression through the points with $^{206}Pb^* > 1\times10^{-13}$ mol yielded the age of 4565.51 ± 0.17 Ma, MSWD = 2.4 (Supplementary Fig. 8). Further increase of the cut-off amount of $^{206}Pb^*$ up to 2×10^{-13} mol yields an isochron with the age of 4565.56 ± 0.12 Ma, MSWD = 1.3 (Supplementary Fig. 9). Using points with a larger amount of $^{206}Pb^* > 5\times10^{-13}$ mol gave a similar result with the age of 4565.55 ± 0.12 Ma, MSWD = 1.5 (Supplementary Fig. 10). In any case, even using data with a low amount of radiogenic Pb does not affect the accuracy significantly. All ages shown in Supplementary Figs. 7-10 are identical within their uncertainties, showing that the choice of cut-off points is not critical. The regression based on the data points with $^{206}Pb^* > 2\times10^{-13}$ mol (Supplementary Fig. 9) appears to be optimal. It yields the best precision and, unlike regressions that include smaller fractions, this isochron shows no dispersion of the points in excess of analytical uncertainties. It does not require further rejections of the points based on other criteria. We thus consider the age of 4565.56 ± 0.12 Ma our best estimate of the crystallization age of EC 002.

Concordance of the U-Pb system

The U-Pb data for fractions W2b and R from analytical session A167 were plotted in a Wetherill-type Concordia diagram (Supplementary Fig. 11). The discordance of the U-Pb system is calculated as $\left(\frac{\frac{207Pb}{206Pb}date - \frac{238U}{206Pb}date}{\frac{207Pb}{206Pb}date}\right)\times 100$. All residue fractions show discordance values from -1.9% to 2.0%, indicating that residues have almost closed the U-Pb system even after acid leaching. The W2b leachates have discordance values of 92-95% for the whole rock and pyroxene fractions, and 63% for plagioclase, indicating the removal of U relative to Pb in this leaching step. All other leachates also have positive discordance values of 8-97% (Supplementary Data 1). Discordia regression for R and W2b fractions (Supplementary Fig. 11) yields an upper concordia intercept at 4565.56 ± 0.34 Ma (corrected for U isotopic composition) which is in agreement with the Pb-Pb isochron age of 4565.56 ± 0.12 Ma within their uncertainties. The zero (within uncertainties) intercept of the Discordia line suggests that fractionation between U and Pb is recent and caused by acid leaching.

Supplementary Discussion

Assessment of reliability of isotopic dates in meteorite chronology

The required level of reliability of the dates of asteroidal and planetary materials for evaluating the distribution of short-lived radionuclides in the Solar System is the same as in the determination of physical constants, e.g., half-lives of radioisotopes, from analysis of natural geological materials [5]. We need to assure that both chronometers date the same event (or synchronous events), that precursor material was completely homogenised during this event, and that the event that is being dated was short compared to the time difference between the formation of the two rocks. The isotopic systems must have remained closed ever since. Both chronometers must yield model-independent and sufficiently precise dates.

The reliability of the dates can be assessed using several criteria, some of which are general, and others are applicable only to certain dating schemes. There are several lines of evidence for closed system behaviour that can be used for any isotope chronometer. The first is the absence of signs of significant metamorphic and/or aqueous processing in the petrologic record. The second is uniformity of the ages of several meteorites or their components that are compositionally similar and probably come from the same parent body (in the case of achondrites), or were produced by the same process in the accreting disk (in the case of CAIs and chondrules). Furthermore, the absence of age variations among similar meteorites with the variable extent of secondary processing (metamorphism, alteration, shock) shows that these secondary processes did not cause detectable migration of parent and daughter isotopes, and hence did not disturb the ages. The absence of excess scattering in isochron regressions also supports closed system behaviour. If the isochron includes data for multiple minerals of igneous origin that plot on the same line, and these minerals have different rates of diffusion of parent and/or daughter elements, then the absence of excess scattering also confirms that the minerals cooled quickly enough to make their closure for diffusion effectively "point-like" [5]. Model independence of the ages is achieved by the high abundance ratio of radiogenic isotopes to non-radiogenic isotopes of the daughter elements. Finally, precision and analytical accuracy rely on the quality of analytical procedures, and sufficient sample size and concentration of the parent and daughter elements to generate precisely measurable radiogenic excesses.

In Pb-isotopic dating, the closed system behaviour, and the accuracy of the dates, are additionally verified by the consistency between $^{206}Pb^*/^{238}U$ and $^{207}Pb^*/^{235}U$ ages (asterisk denotes radiogenic isotopes), which is usually referred to as concordance of the U-Pb system. This criterion is widely used in the dating of young rocks using highly resistant minerals such as zircon where the crystallization age is calculated from the $^{206}Pb^*/^{238}U$ ratio [6]. The ages of very old rocks, including meteorites, are calculated more precisely from the abundance of radiogenic $^{207}Pb^*$ and $^{206}Pb^*$ alone, and variations of the measured $^{206}Pb^*/^{238}U$ and $^{207}Pb^*/^{235}U$ ratios do not necessarily indicate compromised age determination. However, it is important to know when fractionation between U and radiogenic Pb occurred. Present-day fractionation, introduced by laboratory treatment (except for some cases of severe acid leaching [7]) or recent weathering, does not generally cause fractionation of $^{207}Pb^*$ and $^{206}Pb^*$ and age inaccuracy. In contrast, ancient U/Pb fractionation due to asteroidal metamorphism or metasomatism, produces, over time, variable $^{207}Pb^*/^{206}Pb^*$ ratios that complicate Pb-isotopic dating. The time of U/Pb fractionation can be determined from the lower intercept of a regression in a $^{207}Pb^*/^{235}U$ vs. $^{206}Pb^*/^{238}U$ diagram (called concordia diagram). The lower intercept that equals zero within uncertainty indicates that U/Pb fractionation took place at present, and

$^{207}Pb^{*}/^{206}Pb^{*}$ ratios are invariable and accurately reflect the timing of rock crystallization. A non-zero lower intercept points to ancient U/Pb fractionation, or multiple fractionation episodes. Pb-isotopic dates of meteorites that were derived from U-Pb data that yield non-zero lower concordia intercepts should be considered suspect.

Calculation of initial $^{26}Al/^{27}Al$ projected to a certain time

The initial $(^{26}Al/^{27}Al)_0$ ratio of precursor material for each parent body is a function of the Pb-Pb age difference between a reference point and achondrite, and the half-life of ^{26}Al (0.705 Ma, [8]). For better visualization of ^{26}Al heterogeneity in the early Solar System we projected initial $(^{26}Al/^{27}Al)_0$ ratios to the ages of the two commonly used in cosmochemistry references, CAIs (Supplementary Fig. 15A) and volcanic angrite D'Orbigny (Supplementary Fig. 15B). For comparison, we also plot the initial $(^{26}Al/^{27}Al)_0$ of the precursor material for two ungrouped achondrites with carbonaceous affinity, NWA 2976 [9] and NWA 6704 [10], and ungrouped achondrite Asuka 881394 [11].

The uncertainty of the projected initial $^{26}Al/^{27}Al$ includes uncertainties of the $^{26}Al/^{27}Al$ from the isochrons, and uncertainties of the Pb-Pb ages of unknowns. Since change in the age and/or $^{26}Al/^{27}Al$ ratio of the reference points equally shifts all data points, the uncertainties of the reference points are not included. Uncertainty components are combined as quadratic sums, i.e., assuming that they are uncorrelated.

Projections of the initial $(^{26}Al/^{27}Al)_0$ ratio to both reference ages, either CAIs or D'Orbigny, clearly display the heterogeneous distribution of ^{26}Al among achondrites. Thus, the parent body of EC 002 has a CAIs-like initial $(^{26}Al/^{27}Al)_0$ ratio, while precursor materials of other achondrites were formed with a lower $(^{26}Al/^{27}Al)_0$ ratio.

Modelling pre-history of the EC 002 protolith using initial $\delta^{26}Mg^{*}_0$

The intercept of the ^{26}Al-^{26}Mg mineral and whole-rock isochron provides $\delta^{26}Mg^{*}_0=-0.009\pm0.005$ [12]. This initial value suggests that EC 002 was derived from a chondritic source (Supplementary Fig. 16), in agreement with analyses from [13]. A significant increase of the $^{27}Al/^{24}Mg$ ratio up to 1.299 (calculated from data [13]) from 0.101 (CI value, [14]) happened rapidly after accretion during partial melting of the magma. The isotopic evolution of $^{26}Mg^{*}$ (Supplementary Fig. 16) shows that the time of accretion of the EC 002 parent body is not resolved from the EC 002 crystallization time, unlike the case shown in NWA 7325 [15].

Supplementary Figures

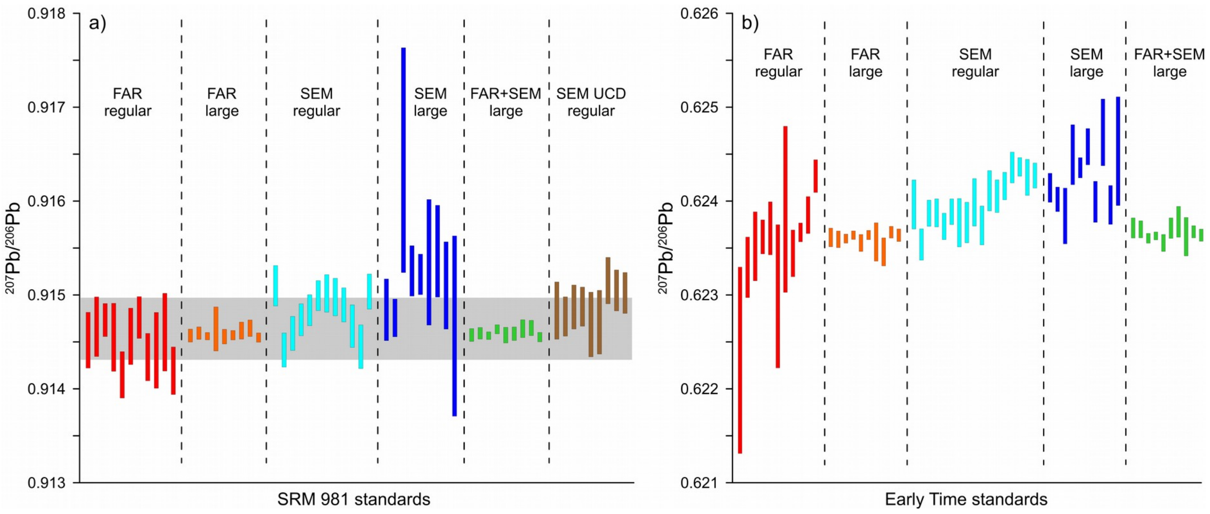

Supplementary Fig. 1.
Summary of $^{207}Pb/^{206}Pb$ ratios for the regular and large SRM 981 (a) and Early Time (b) standards. Shaded area is certified $^{207}Pb/^{206}Pb$ value of 0.91464 ± 0.00033 for the SRM 981. Source data are provided as a Source Data file. Error bars are 2σ.

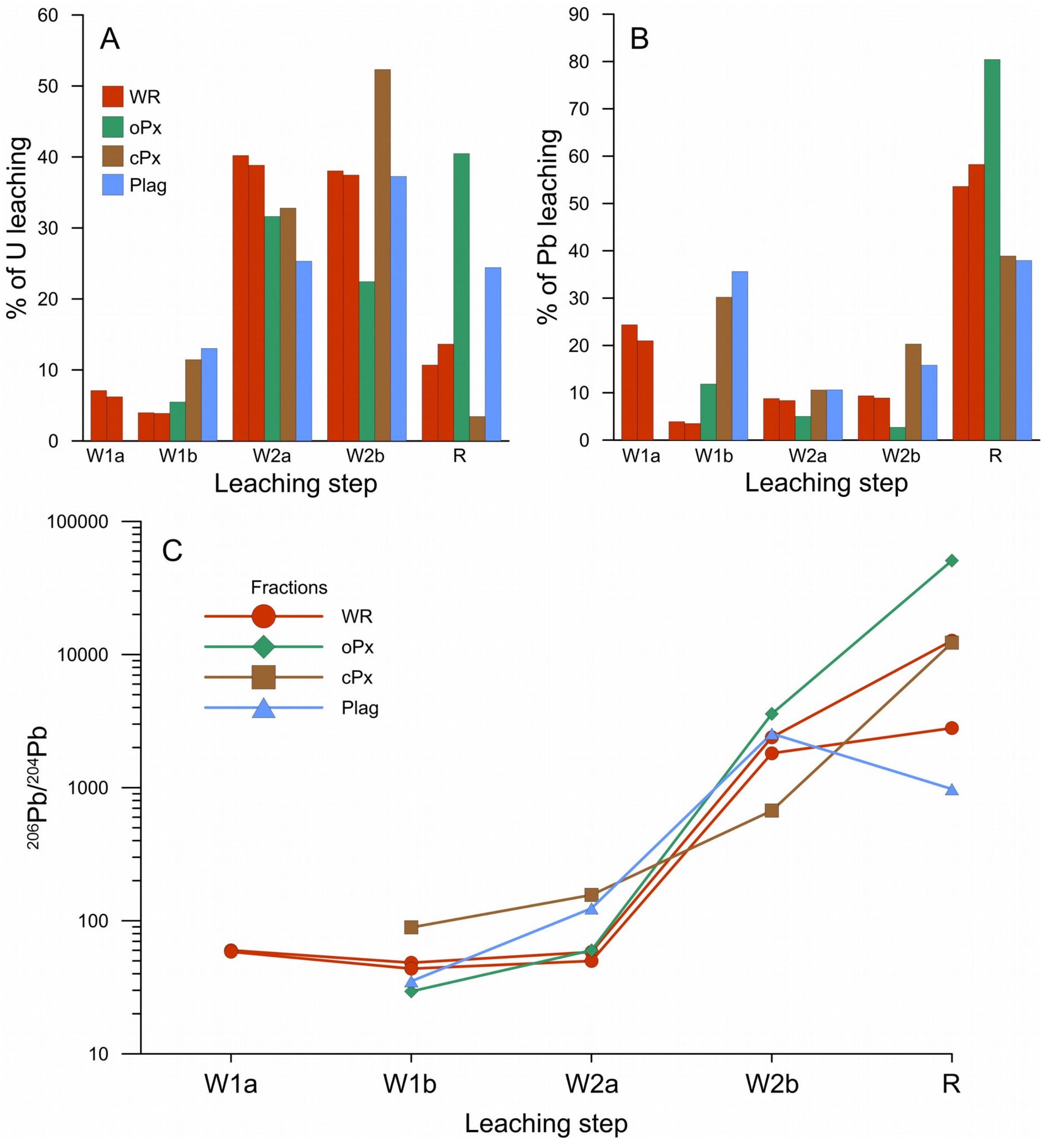

Supplementary Fig. 2.
Uranium and Pb distribution (A and B) and $^{206}Pb/^{204}Pb$ ratios (C) between leaching steps. WR – whole rock (red), oPx – orthopyroxene (green), cPx – clinopyroxene (brown), plag – plagioclase (blue).

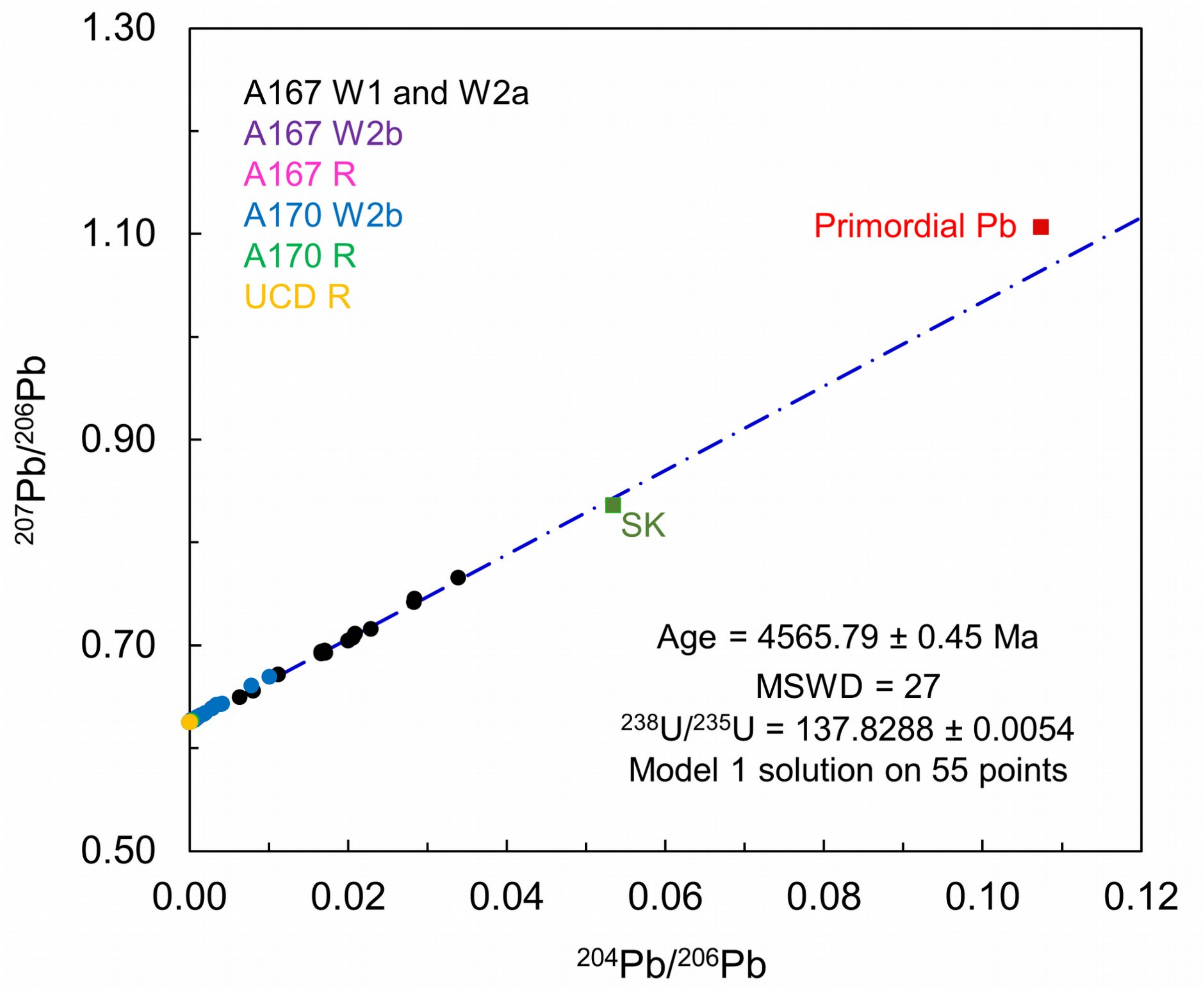

Supplementary Fig. 3.
Pb-Pb isochron (dot-dash blue line) diagram for all analysed fractions (circles). Primordial Pb (red square) – isotopic composition of Pb in Canyon Diablo troilite [16], SK (green square) – isotopic composition of modern terrestrial Pb [17]. Symbols are larger than error ellipses.

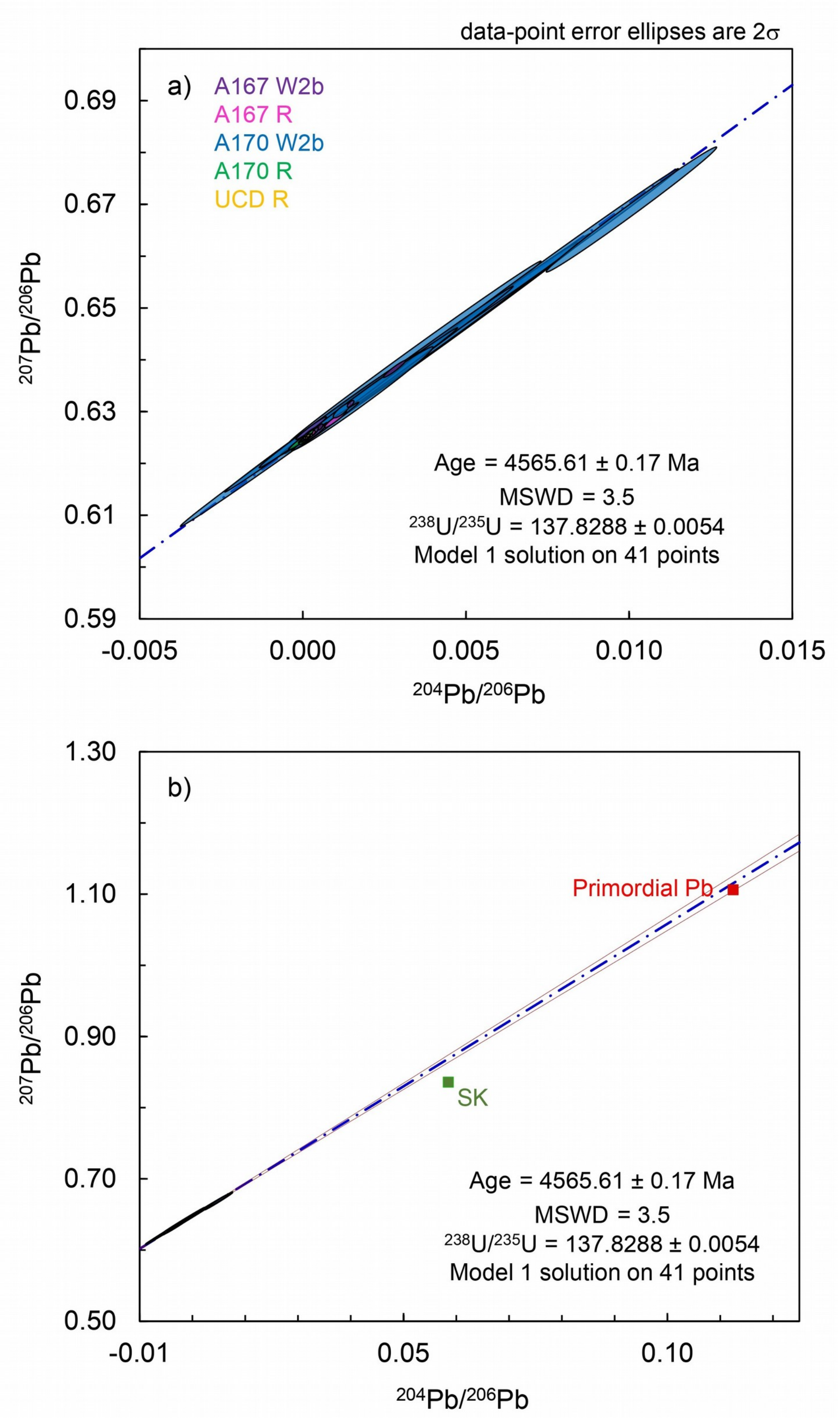

Supplementary Fig. 4.
A – Pb-Pb isochron (dot-dash blue line) diagram for W2b and R fractions. B – the same isochron (red lines are error envelope) passes through the isotopic composition of the primordial Pb (red square) [16,18]. SK (green square) – isotopic composition of modern terrestrial Pb [17]. Error ellipses are 2σ.

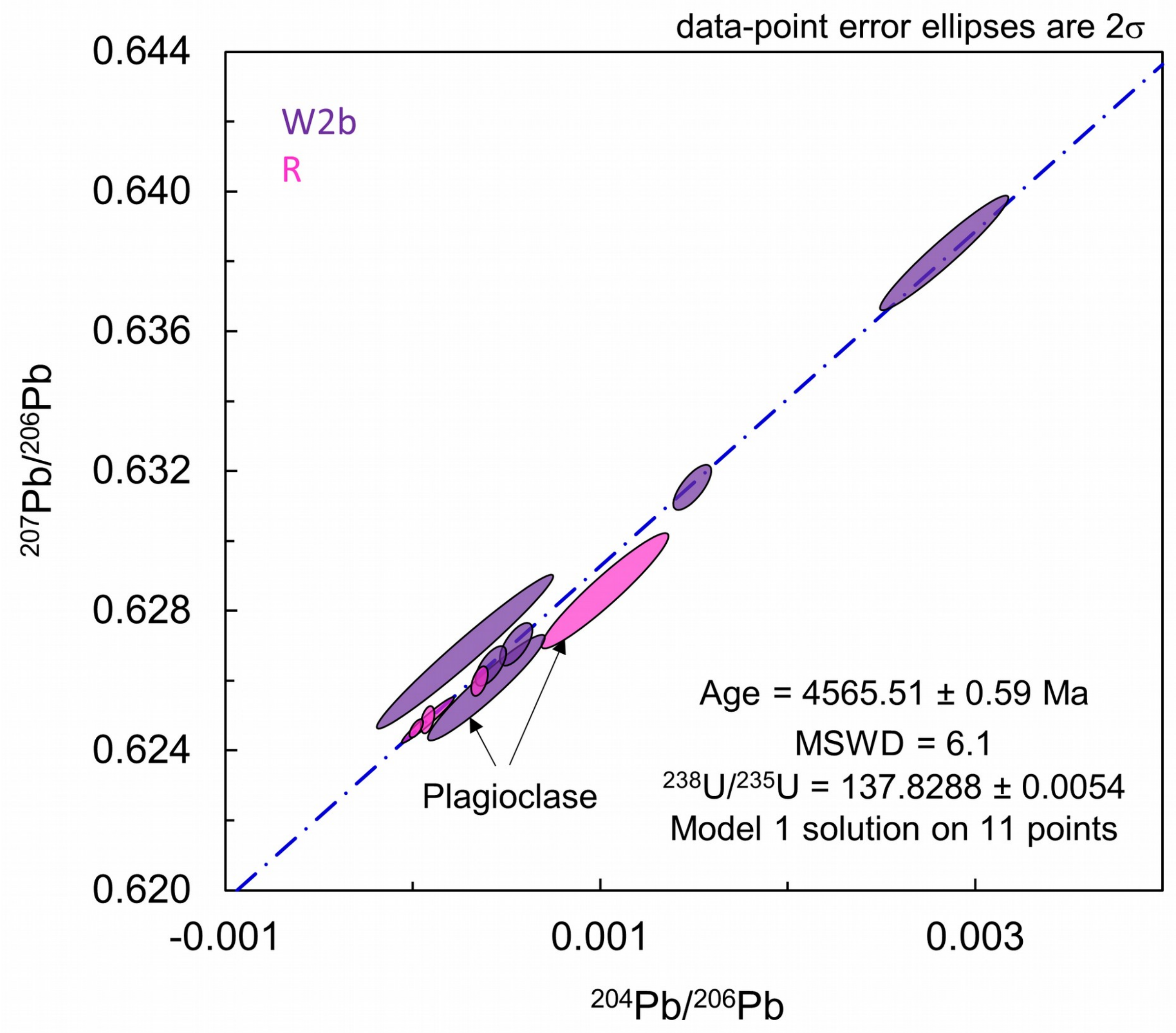

Supplementary Fig. 5.
Pb-Pb isochron (dot-dash blue line) diagram for R (pink) and W2b (purple) fractions from A167 analytical session. Data points from the A170 and UCD sessions are not shown for clarity. All plagioclase points lie below the isochron, suggesting that the U-Pb system was open. The data point above the isochron is W2b leachate of pyroxene, its exclusion is described further. Error ellipses are 2σ.

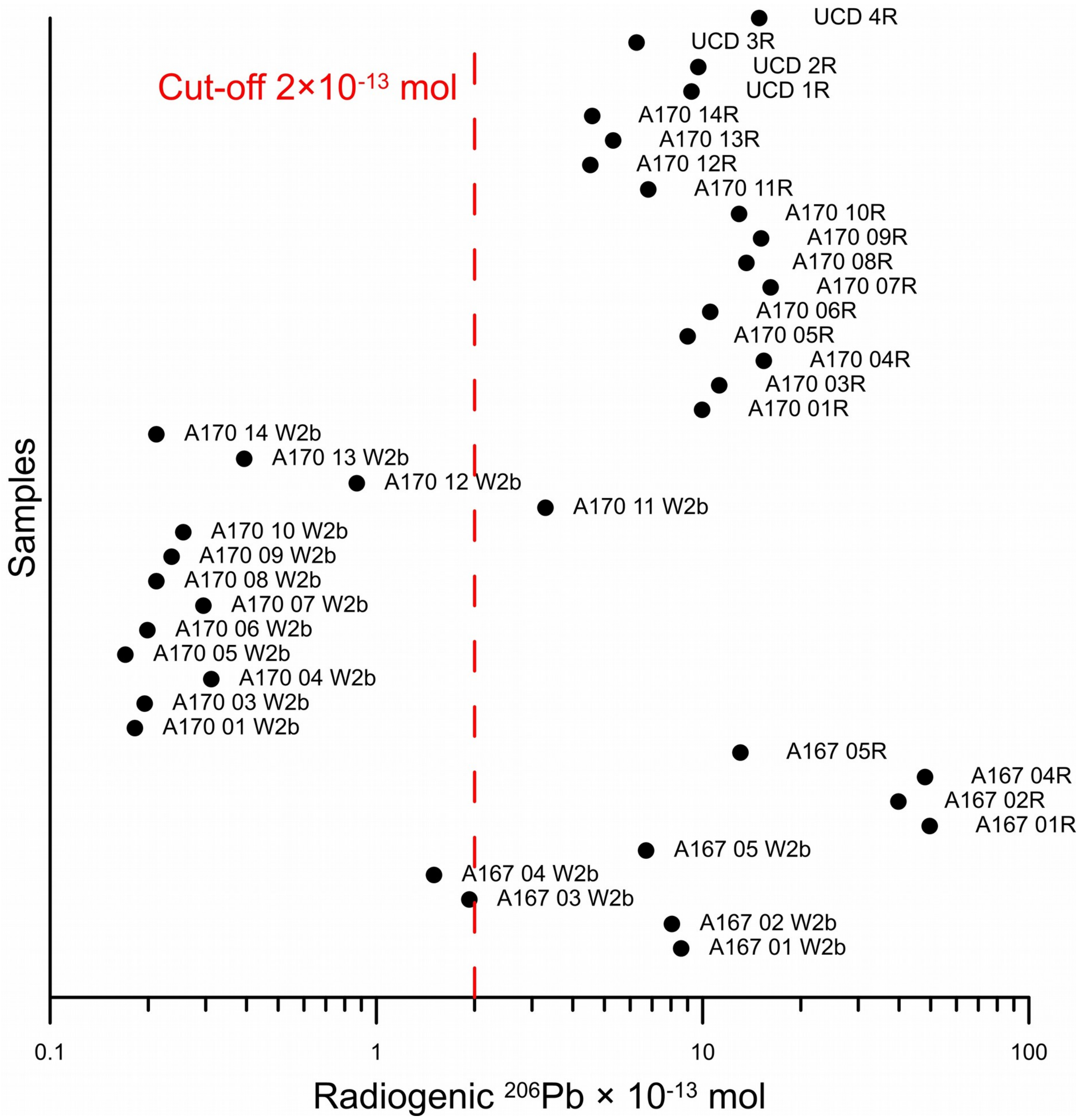

Supplementary Fig. 6.
The distribution of radiogenic ^{206}Pb in W2b and R fractions in A167, A170, and UCD analytical sessions.

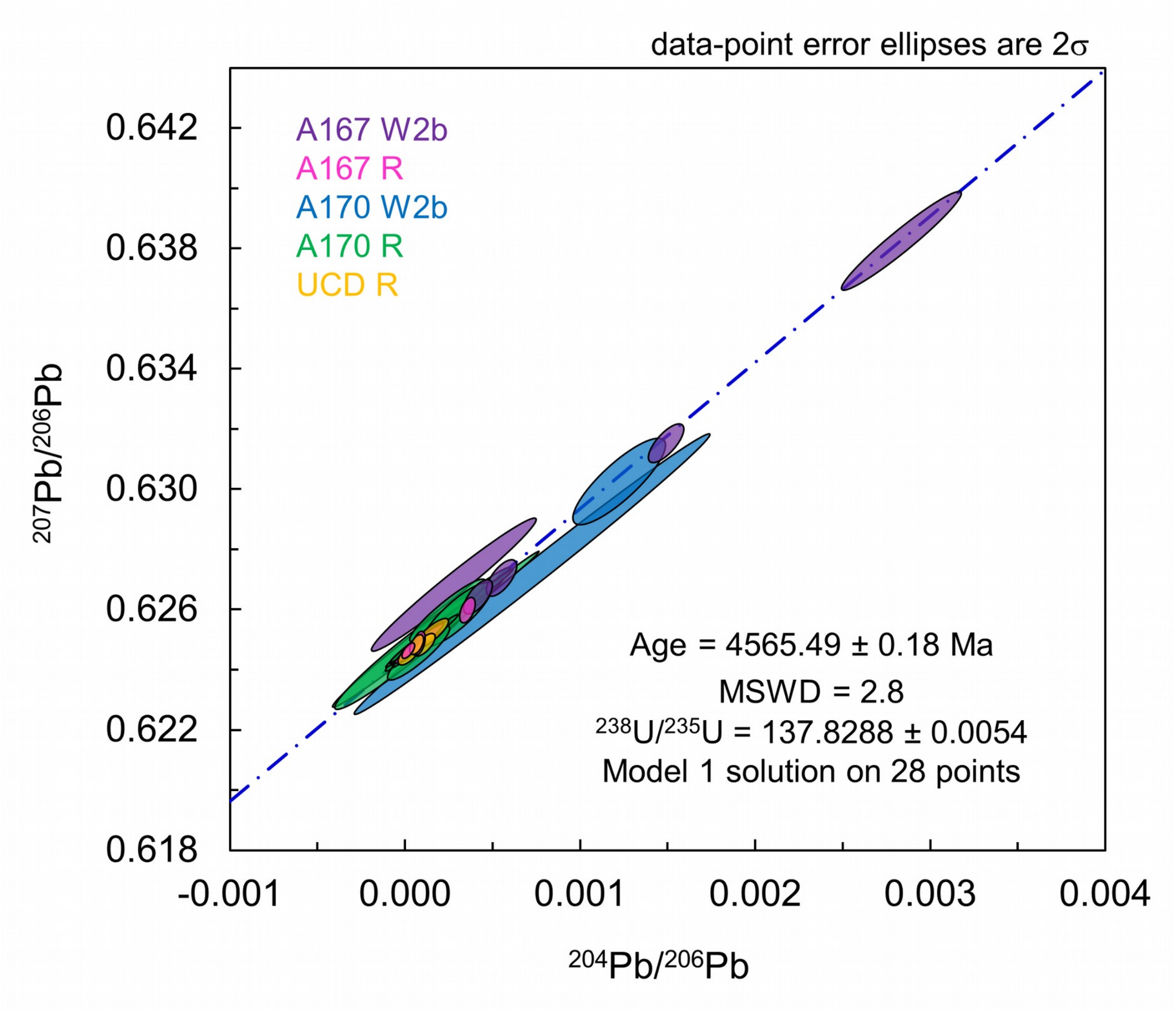

Supplementary Fig. 7.
Pb-Pb isochron (dot-dash blue line) diagram for R and W2b fractions with $^{206}Pb^* > 0.5\times10^{-13}$ mol. Error ellipses are 2σ.

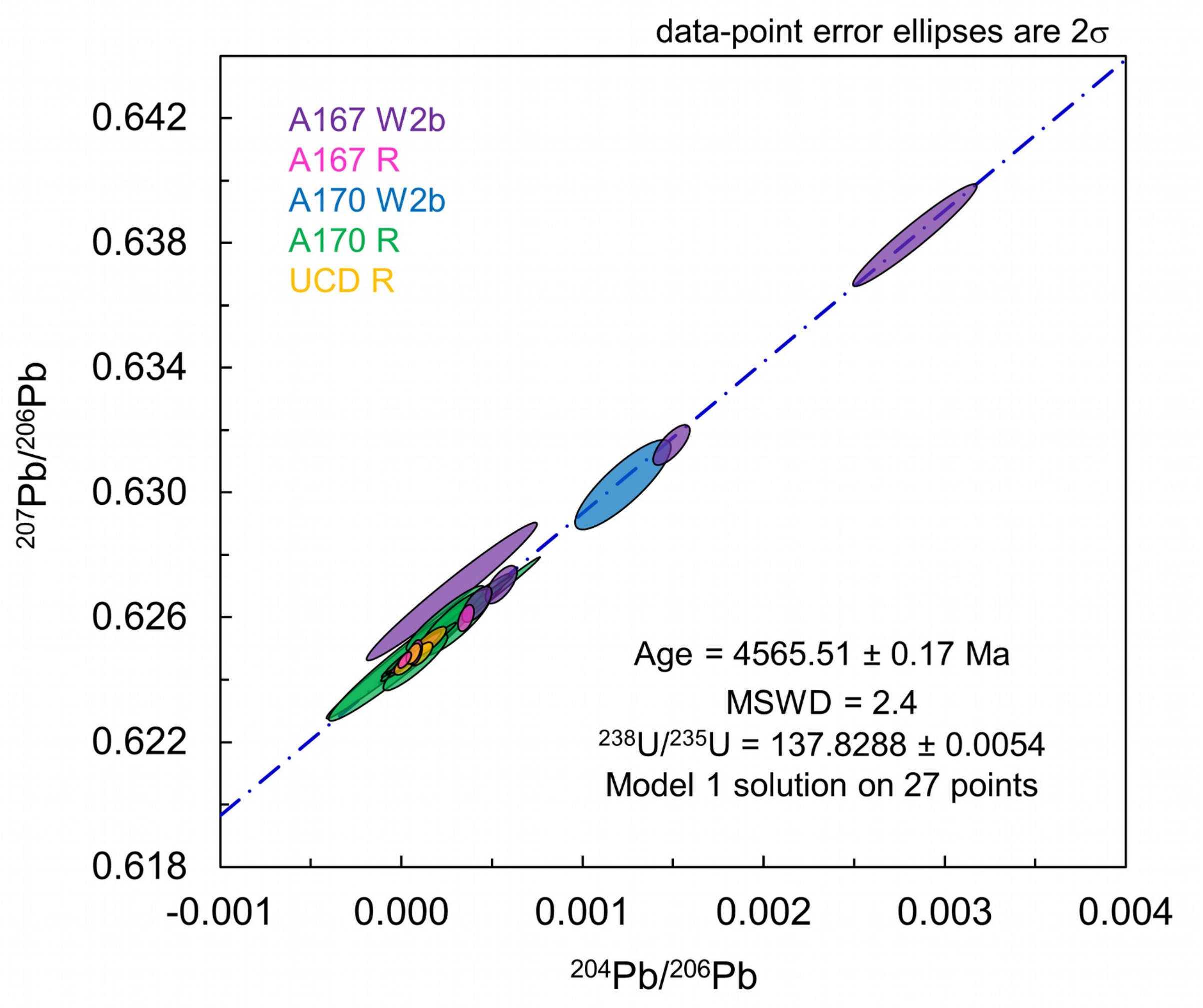

Supplementary Fig. 8.
Pb-Pb isochron (dot-dash blue line) diagram for R and W2b fractions with $^{206}Pb^* > 1.0\times10^{-13}$ mol. Error ellipses are 2σ.

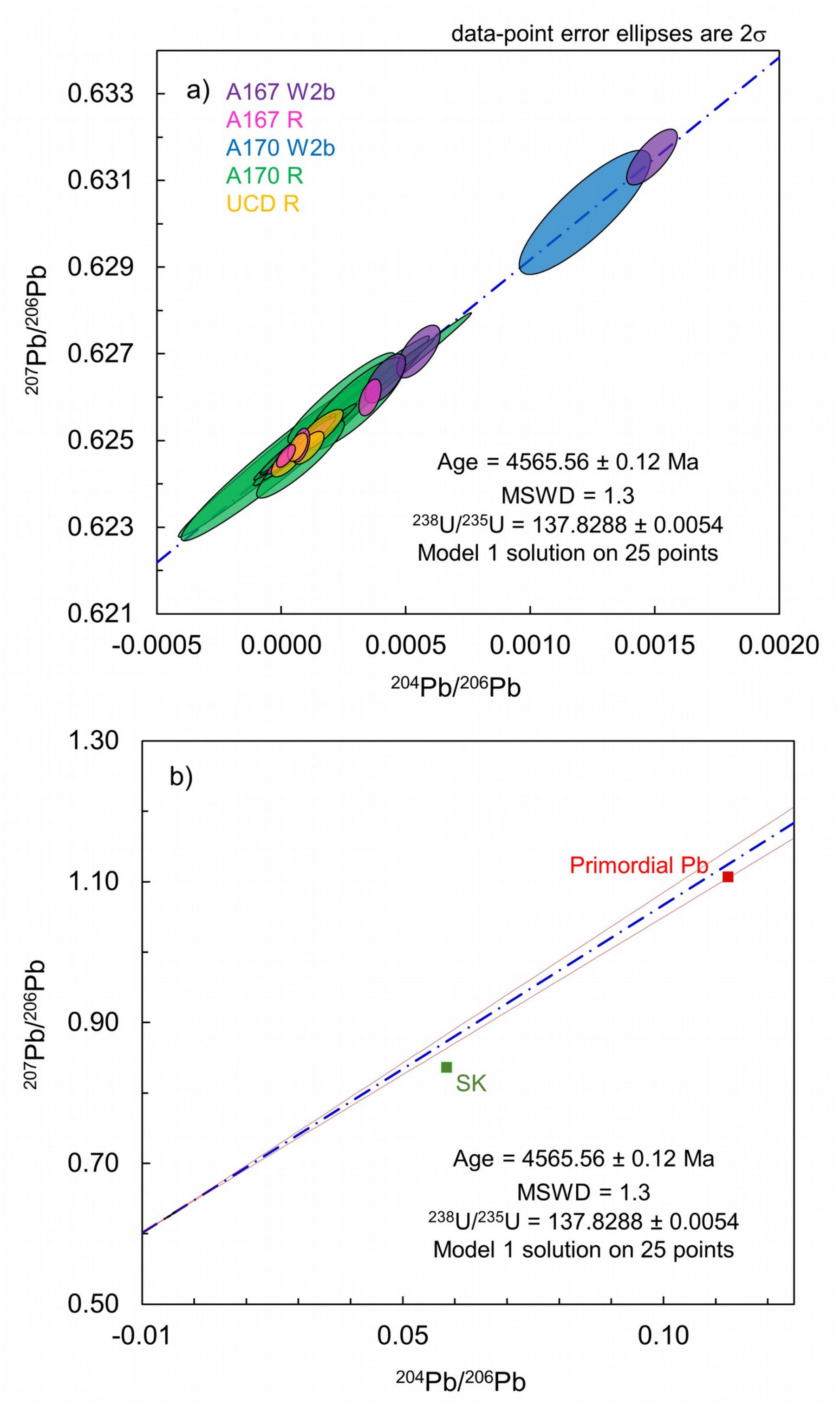

Supplementary Fig. 9.
A - Pb-Pb isochron (dot-dash blue line) diagram for R and W2b fractions with $^{206}Pb^* > 2.0\times10^{-13}$ mol. B – the same isochron (red lines are error envelope) passes through the isotopic composition of the primordial Pb (red square) [16,18]. SK (green square) – isotopic composition of modern terrestrial Pb [17]. Error ellipses are 2σ.

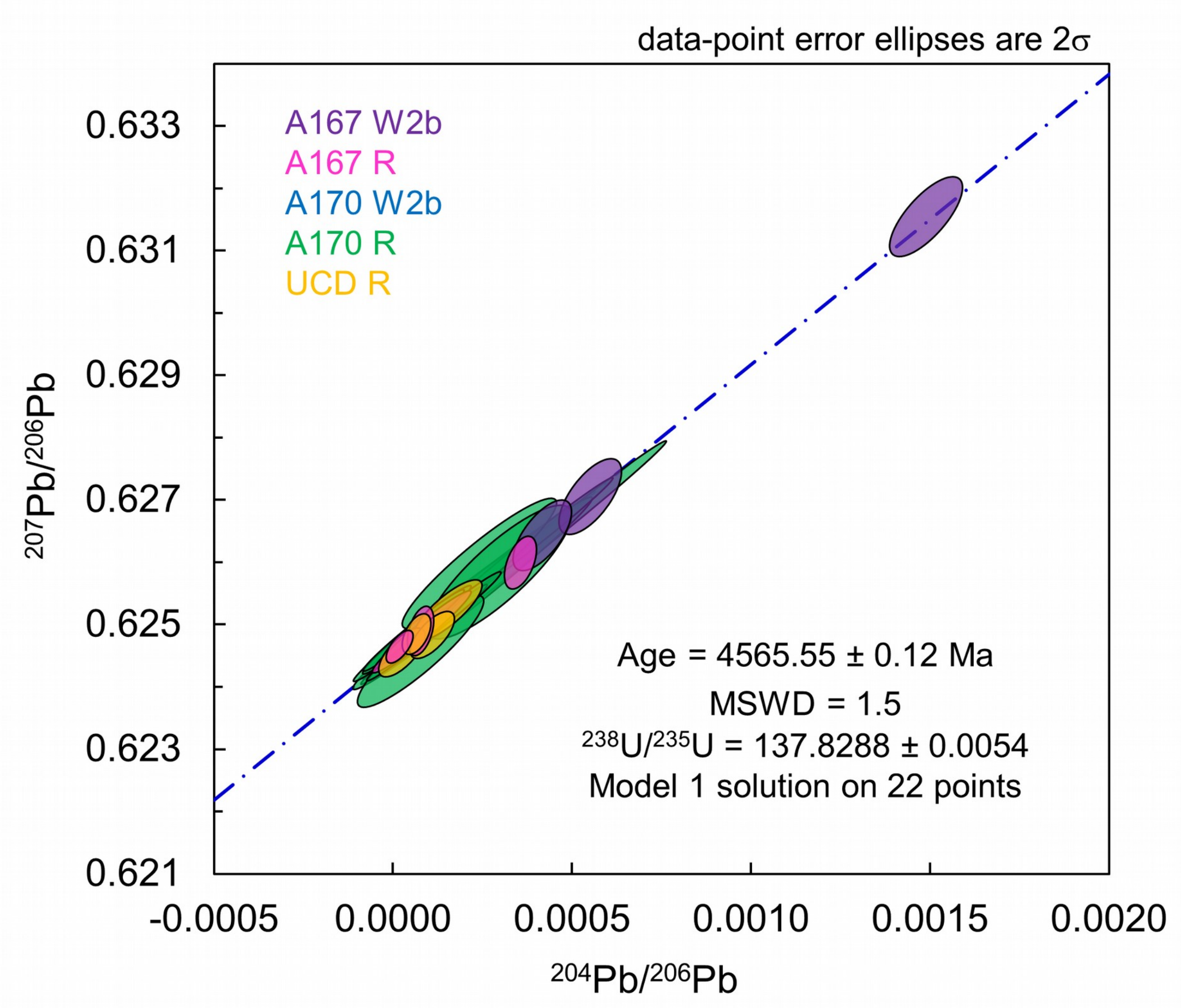

Supplementary Fig. 10.
Pb-Pb isochron (dot-dash blue line) diagram for R and W2b fractions with ^{206}Pb* > 5.0×10^{-13} mol. Error ellipses are 2σ.

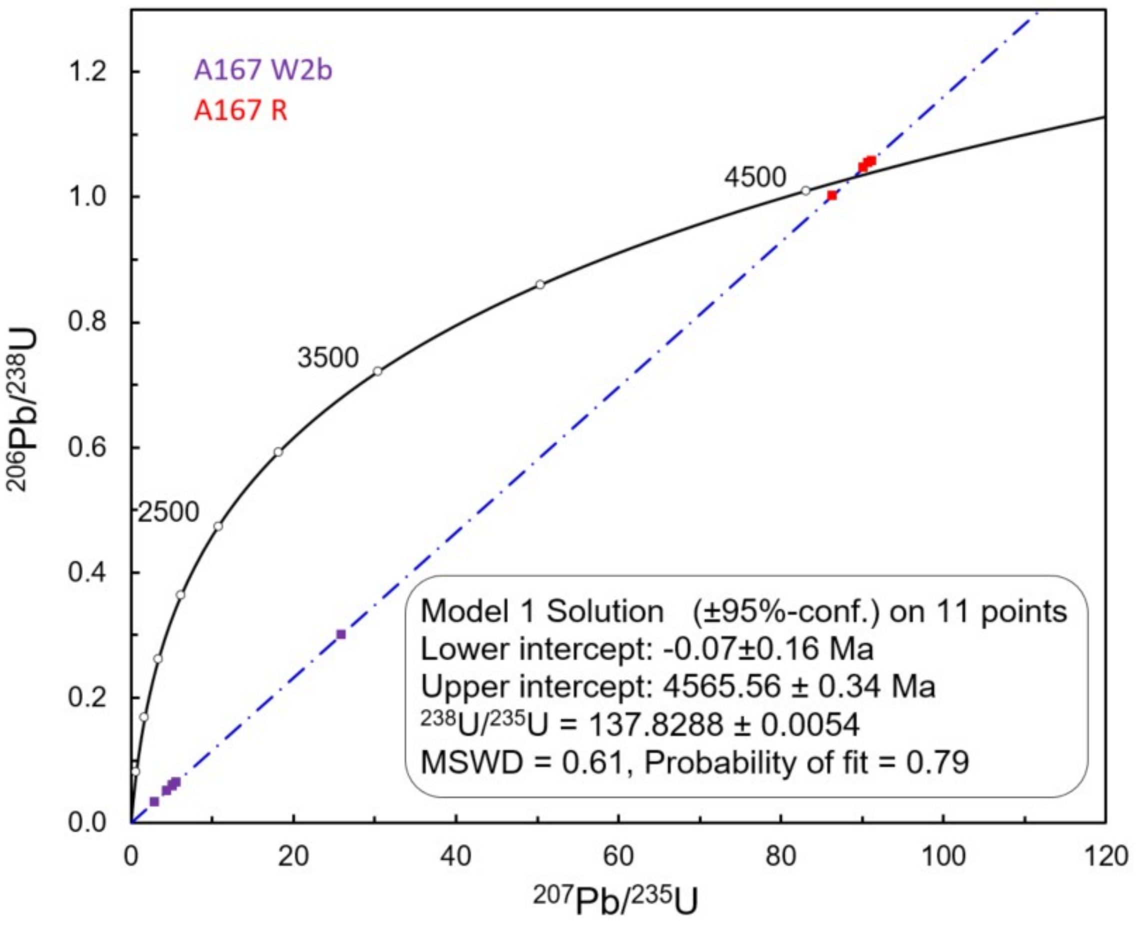

Supplementary Fig. 11.
A167 W2b and R data plotted in Concordia diagram. The dot-dash blue line represents the Discordia line. The intercept at zero confirms that U-Pb disturbance was caused by our leaching procedure and before U-Pb isotopic system was closed. An upper interception with an age of 4565.56 ± 0.34 Ma is in agreement with Pb-Pb isochron and $^{207}Pb^*/^{206}Pb^*$ model ages, confirming their accuracy.

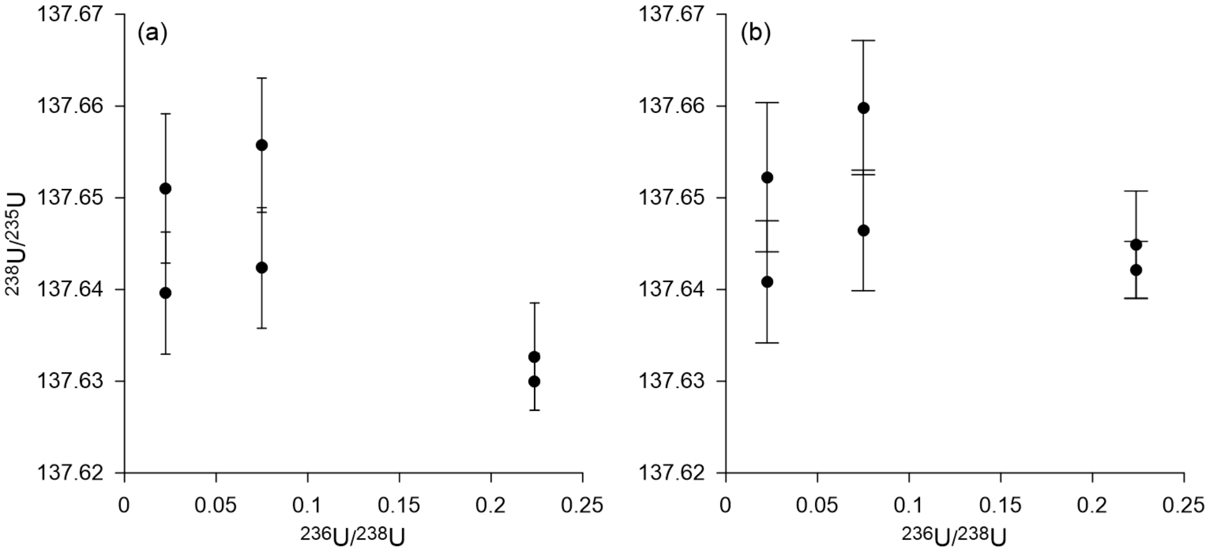

Supplementary Fig. 12.
Correlation of the $^{238}U/^{235}U$ and $^{236}U/^{238}U$ ratios in the different sample/spike mixtures using certified (a) and measured (b) isotopic composition of the double spike IRMM-3636. Source data are provided as a Source Data file. Error bars are 2σ.

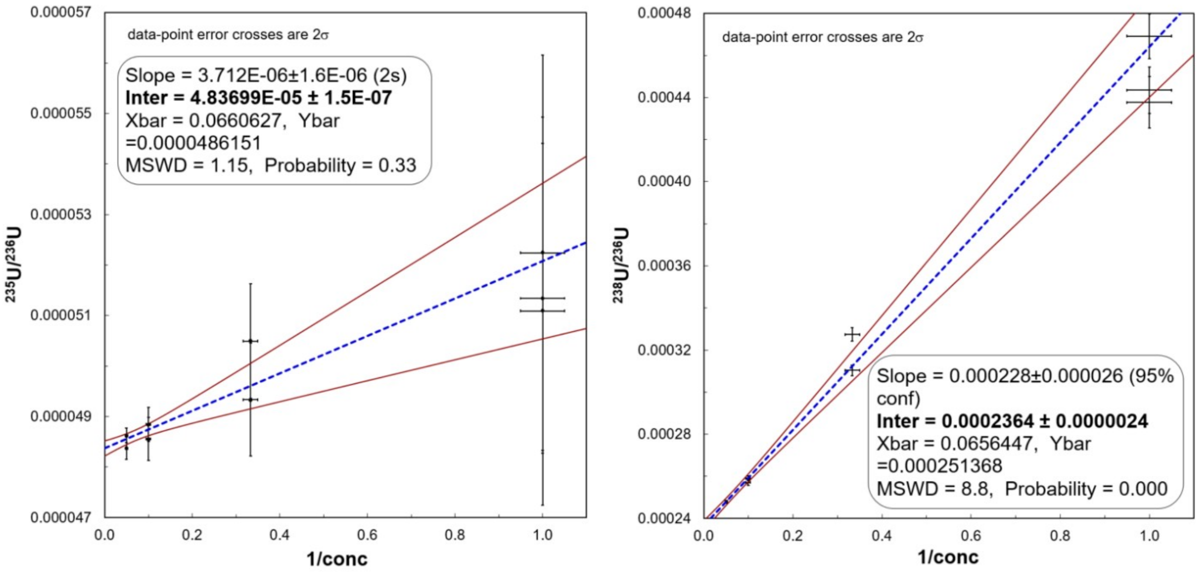

Supplementary Fig. 13.
Isotopic composition ($^{235}U/^{236}U$ and $^{238}U/^{236}U$ ratios) of the IRMM-3636 double spike. The dash blue line is the regression line, red lines are error envelopes. Source data are provided as a Source Data file. Error bars are 2σ.

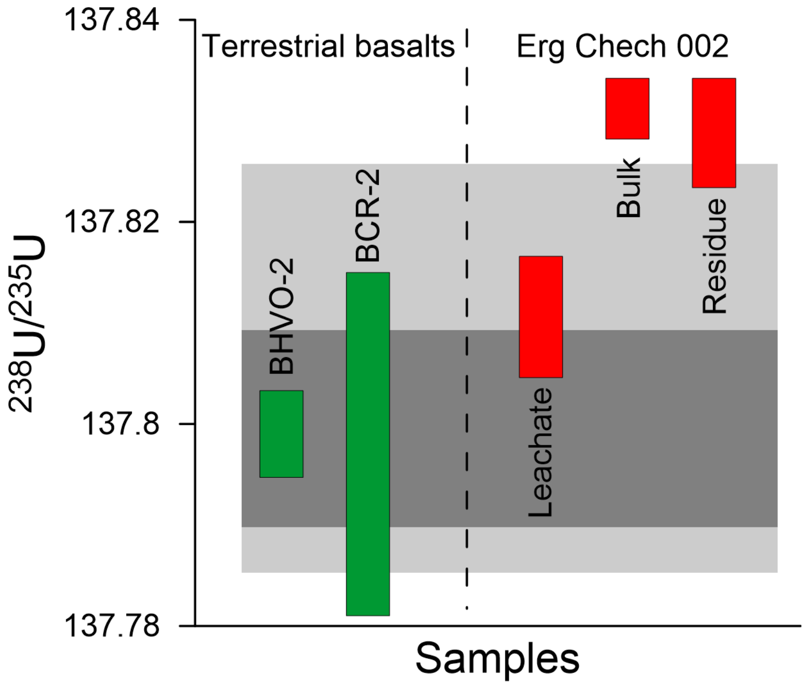

Supplementary Fig. 14.
Uranium isotopic composition of EC 002 relative to terrestrial basalts (this study), and average Solar system values of 137.786±0.013 [19] (dark grey) and 137.794±0.027 [20] (light grey). Error bars are 2σ.

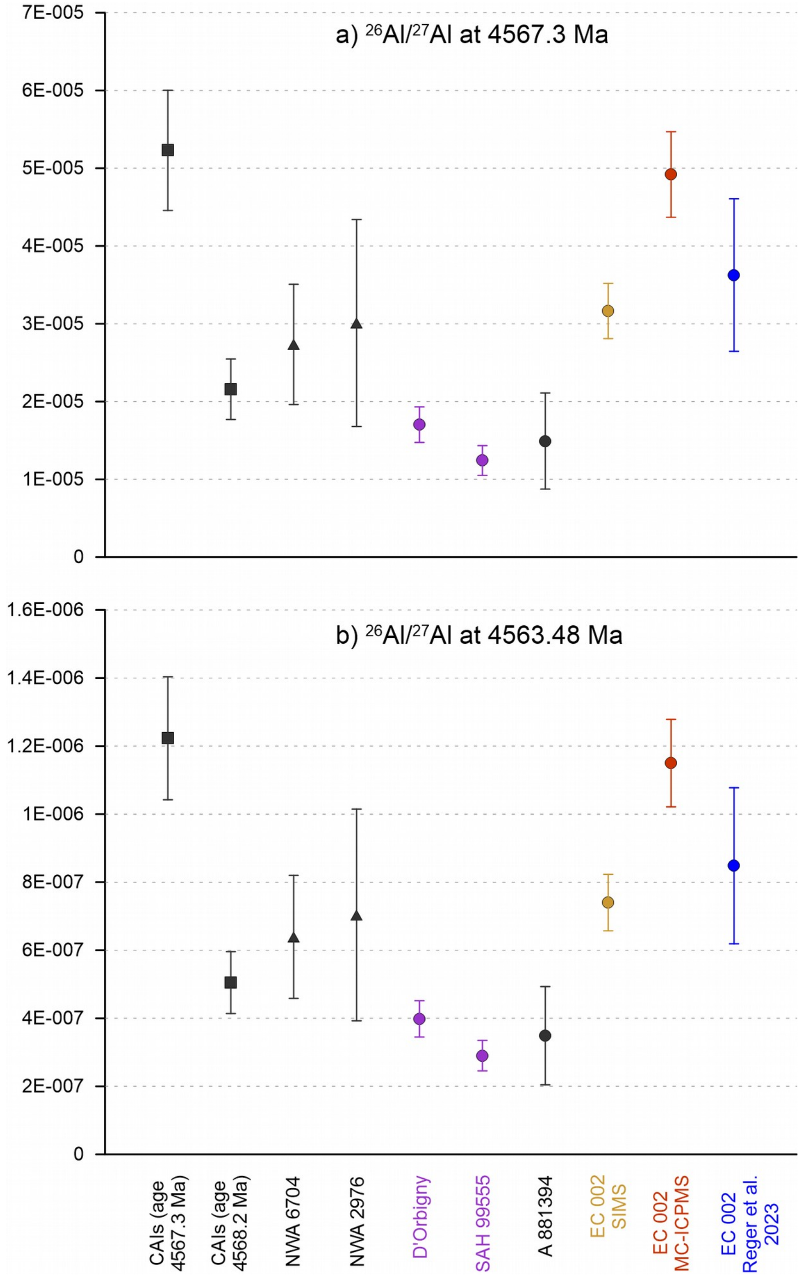

Supplementary Fig. 15.
Initial $^{26}Al/^{27}Al$ ratios for CAIs [21] (squares), volcanic angrites [22] (purple circles) and ungrouped achondrites [9,11,23], projected to either to the age of CAIs (a) or the age of D'Orbigny (b). Achondrites with carbonaceous affinity are shown with triangles, non-carbonaceous achondrites – with circles. The values for Erg Chech 002 are shown for SIMS data [13] (light brown) and MC-ICPMS data [12] (red) using the Pb-Pb age from this study, and data from [24] (blue). The age of D'Orbigny is calculated from the Pb-Pb isochron age of 4564.42±0.12 Ma (@$^{238}U/^{235}U$=137.88) from [2], and $^{238}U/^{235}U$=137.7906±0.0060, a weighted average of four measurements from [25,26]. The age of Sahara 99555 is calculated from the Pb-Pb ages of 4564.61±0.13 Ma (@$^{238}U/^{235}U$=137.88), a weighted average of Pb-Pb isochron ages from [3,27], and $^{238}U/^{235}U$=137.7945±0.0095, a weighted average of two measurements from [19,26]. Source data are provided as a Source Data file. Error bars include errors (2σ) from Al-Mg and Pb-Pb data.

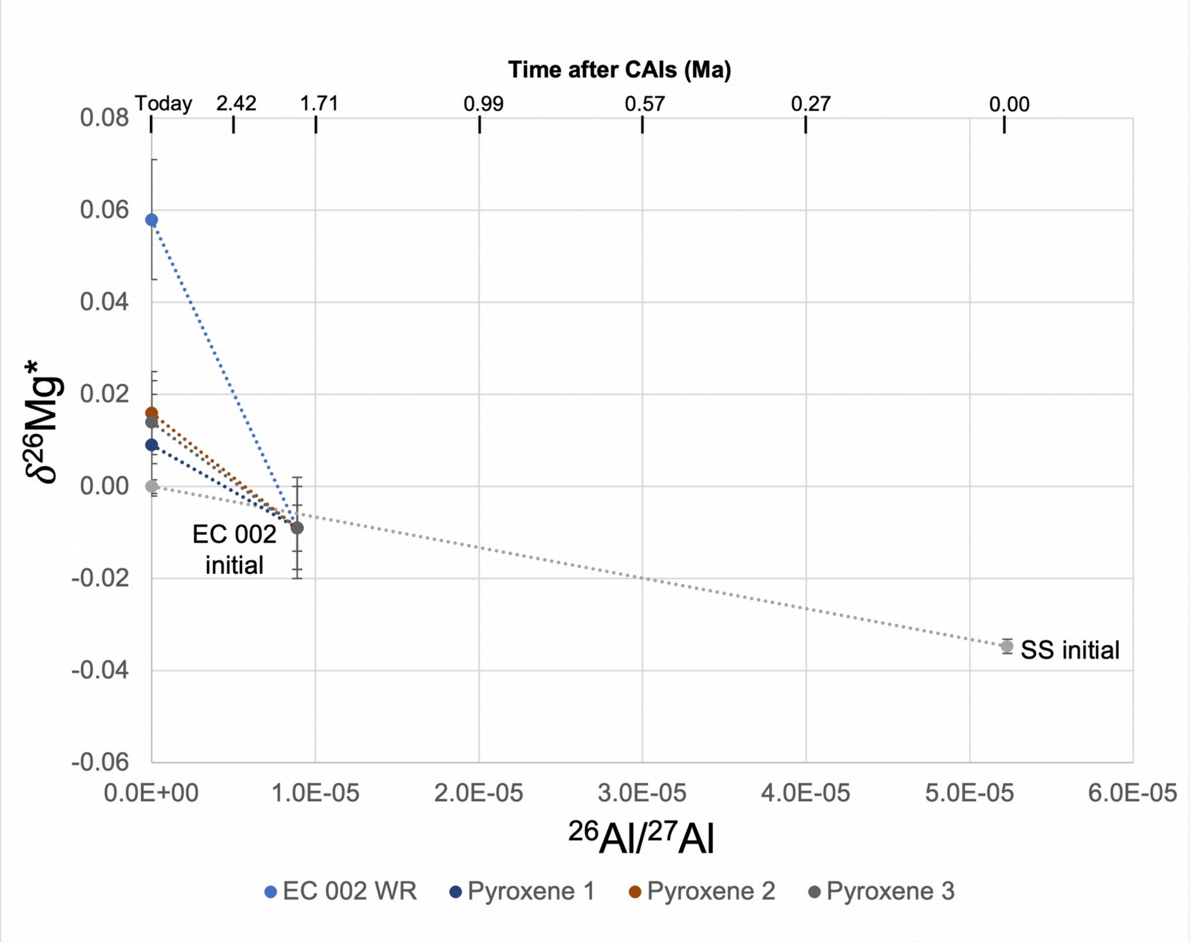

Supplementary Fig. 16.
^{26}Mg* isotopic evolution diagram for EC 002 showing the radiogenic ingrowth of ^{26}Mg* from ^{26}Al decay in whole-rock and pyroxene fractions. The Solar System initial composition (SS initial) from [21], SS initial evolution (grey dotted line) corresponds to solar (=CI) $^{27}Al/^{24}Mg$=0.101 from [14]. EC 002 initial and δ^{26}Mg* data for whole-rock (WR) and pyroxene fractions are from [12]. Source data are provided as a Source Data file. Error bars are 2σ.

Supplementary Tables

Supplementary Table 1.

Uranium isotopic composition of the standards, terrestrial basalts, and EC 002

Sample	$^{238}U/^{235}U$ normalized	95% MAD	n	MSWD	Published value		Reference
IRMM-184	137.6828	0.0027	32	5	137.6830	0.0200	[28]
SRM 960	137.8505	0.0061	20	6.5	137.8370	0.0150	[28]
BHVO-2	137.7990	0.0043	3	0.5	137.7940	0.0030	[29]
BCR-2	137.7980	0.0170	3	4.9	137.8020	0.0030	[29]
EC 002 Bulk	137.8312	0.0030	4	0.8			
EC 002 Wash	137.8106	0.0060	1				
EC 002 Residue	137.8288	0.0054	5	2.1			

Supplementary Table 2.

Cr isotopic composition of Erg Chech 002

Sample	$\varepsilon^{53}Cr$	±2SE	$\varepsilon^{54}Cr$	±2SE
Erg Chech 002 (A)	0.36	0.03	-0.70	0.07
Erg Chech 002 (B)	0.39	0.04	-0.59	0.07
Average	0.37	0.02	-0.65	0.10

Supplementary Table 3.

Sr isotope systematics of Erg Chech 002

Sample	$^{87}Sr/^{86}Sr$	± 2SE	$^{84}Sr/^{86}Sr$	± 2SE	$\varepsilon^{84}Sr$	± 2SE	Comment
A167 01R	0.7402478	0.0000035	0.0564895	0.0000036	-0.303	0.635	WR
A167 02R	0.7407883	0.0000034	0.0564925	0.0000021	0.223	0.364	WR
A167 03R	0.7733258	0.0000055	0.0564892	0.0000026	-0.368	0.460	Px
A167 04R	0.7824441	0.0000040	0.0564840	0.0000049	-1.273*	0.864*	Px
A167 05R	0.7443510	0.0000040	0.0564919	0.0000022	0.124	0.394	Px
A167 06R	0.7523631	0.0000041	0.0564917	0.0000024	0.077	0.422	Plag
A169 43**	0.7381390	0.0000019	0.0564923	0.0000020	0.188	0.355	WR
Weighted average					0.060	0.240	

* value excluded from weighted average calculation because of low precision

** fraction from U IC ($^{238}U/^{235}U$) session

Supplementary Table 4.

Oxygen isotope data of Erg Chech 002 (University of Göttingen, UCLA, and UNM data)

Sample	Comment	N	$\delta'^{17}O$	2SD	2SEM	$\delta'^{18}O$	2SD	2SEM	$\Delta'^{17}O$	2SD	2SEM
EC 002	UG, this study	7	1.875	0.406	0.154	3.769	0.743	0.281	-0.114	0.023	0.009
EC 002	UNM, [30]	7	1.720	0.202	0.076	3.497	0.389	0.147	-0.126	0.030	0.011
EC 002	UCLA*, this study	1	2.251	0.009		4.629	0.004		-0.193	0.009	
EC 002	UCLA, this study	1	2.299	0.009		4.629	0.004		-0.145	0.009	
SC olivine	UG, this study	10	2.700	0.203	0.064	5.212	0.391	0.124	-0.052	0.013	0.004

*UCLA data before anchoring to the composition of SC olivine

Supplementary References

1 Shannon, R. D. Revised effective ionic radii and systematic studies of interatomic distances in halides and chalcogenides. *Acta Crystallographica Section A* **32**, 751-767, doi:https://doi.org/10.1107/S0567739476001551 (1976).

2 Amelin, Y. U–Pb ages of angrites. *Geochimica et Cosmochimica Acta* **72**, 221-232, doi:https://doi.org/10.1016/j.gca.2007.09.034 (2008).

3 Amelin, Y. The U–Pb systematics of angrite Sahara 99555. *Geochimica et Cosmochimica Acta* **72**, 4874-4885, doi:https://doi.org/10.1016/j.gca.2008.07.008 (2008).

4 Ito, K. T. M., Hibiya, Y., Homma, Y., Mikouchi, T. & Iizuka, T. The promise and potential pitfalls of acid leaching for PbPb chronology. *Chemical Geology* **525**, 343-355, doi:https://doi.org/10.1016/j.chemgeo.2019.07.035 (2019).

5 Begemann, F. *et al.* Call for an improved set of decay constants for geochronological use. *Geochimica et Cosmochimica Acta* **65**, 111-121, doi:https://doi.org/10.1016/S0016-7037(00)00512-3 (2001).

6 Schoene, B. in *Treatise on Geochemistry (Second Edition)* (ed Karl K. Turekian) 341-378 (Elsevier, 2014).

7 Amelin, Y. *et al.* in *Goldschmidt Conference Abstracts 57.*

8 Norris, T. L., Gancarz, A. J., Rokop, D. J. & Thomas, K. W. Half-life of 26Al. *Journal of Geophysical Research: Solid Earth* **88**, B331-B333, doi:https://doi.org/10.1029/JB088iS01p0B331 (1983).

9 Bouvier, A., Spivak-Birndorf, L. J., Brennecka, G. A. & Wadhwa, M. New constraints on early Solar System chronology from Al–Mg and U–Pb isotope systematics in the unique basaltic achondrite Northwest Africa 2976. *Geochimica et Cosmochimica Acta* **75**, 5310-5323, doi:https://doi.org/10.1016/j.gca.2011.06.033 (2011).

10 Amelin, Y. *et al.* U-Pb, Rb-Sr and Ar-Ar systematics of the ungrouped achondrites Northwest Africa 6704 and Northwest Africa 6693. *Geochimica et Cosmochimica Acta* **245**, 628-642, doi:https://doi.org/10.1016/j.gca.2018.09.021 (2019).

11 Wimpenny, J. *et al.* Reassessing the origin and chronology of the unique achondrite Asuka 881394: Implications for distribution of 26Al in the early Solar System. *Geochimica et Cosmochimica Acta* **244**, 478-501, doi:https://doi.org/10.1016/j.gca.2018.10.006 (2019).

12 Fang, L. *et al.* Half-life and initial Solar System abundance of 146Sm determined from the oldest andesitic meteorite. *Proceedings of the National Academy of Sciences* **119**, e2120933119, doi:doi:10.1073/pnas.2120933119 (2022).

13 Barrat, J.-A. *et al.* A 4,565-My-old andesite from an extinct chondritic protoplanet. *Proceedings of the National Academy of Sciences* **118**, e2026129118, doi:10.1073/pnas.2026129118 (2021).

14 Lodders, K. Solar System Abundances and Condensation Temperatures of the Elements. *The Astrophysical Journal* **591**, 1220-1247, doi:10.1086/375492 (2003).

15 Koefoed, P. *et al.* U–Pb and Al–Mg systematics of the ungrouped achondrite Northwest Africa 7325. *Geochimica et Cosmochimica Acta* **183**, 31-45, doi:https://doi.org/10.1016/j.gca.2016.03.028 (2016).

16 Tatsumoto, M., Knight, R. J. & Allegre, C. J. Time Differences in the Formation of Meteorites as Determined from the Ratio of Lead-207 to Lead-206. *Science* **180**, 1279-1283, doi:10.1126/science.180.4092.1279 (1973).

17 Stacey, J. S. & Kramers, J. D. Approximation of terrestrial lead isotope evolution by a two-stage model. *Earth and Planetary Science Letters* **26**, 207-221, doi:https://doi.org/10.1016/0012-821X(75)90088-6 (1975).
18 Blichert-Toft, J., Zanda, B., Ebel, D. S. & Albarède, F. The Solar System primordial lead. *Earth and Planetary Science Letters* **300**, 152-163, doi:https://doi.org/10.1016/j.epsl.2010.10.001 (2010).
19 Connelly, J. N. *et al.* The absolute chronology and thermal processing of solids in the solar protoplanetary disk. *Science* **338**, 651-655 (2012).
20 Goldmann, A., Brennecka, G., Noordmann, J., Weyer, S. & Wadhwa, M. The uranium isotopic composition of the Earth and the Solar System. *Geochimica et Cosmochimica Acta* **148**, 145-158, doi:https://doi.org/10.1016/j.gca.2014.09.008 (2015).
21 Jacobsen, B. *et al.* 26Al–26Mg and 207Pb–206Pb systematics of Allende CAIs: Canonical solar initial 26Al/27Al ratio reinstated. *Earth and Planetary Science Letters* **272**, 353-364, doi:https://doi.org/10.1016/j.epsl.2008.05.003 (2008).
22 Schiller, M., Connelly, J. N., Glad, A. C., Mikouchi, T. & Bizzarro, M. Early accretion of protoplanets inferred from a reduced inner solar system 26Al inventory. *Earth and Planetary Science Letters* **420**, 45-54, doi:https://doi.org/10.1016/j.epsl.2015.03.028 (2015).
23 Sanborn, M. E. *et al.* Carbonaceous achondrites Northwest Africa 6704/6693: Milestones for early Solar System chronology and genealogy. *Geochimica et Cosmochimica Acta* **245**, 577-596, doi:https://doi.org/10.1016/j.gca.2018.10.004 (2019).
24 Reger, P. M. *et al.* Al-Mg and U-Pb chronological records of Erg Chech 002 ungrouped achondrite meteorite. *Geochimica et Cosmochimica Acta* **343**, 33-48, doi:https://doi.org/10.1016/j.gca.2022.12.025 (2023).
25 Brennecka, G. A. & Wadhwa, M. Uranium isotope compositions of the basaltic angrite meteorites and the chronological implications for the early Solar System. *Proceedings of the National Academy of Sciences* **109**, 9299-9303 (2012).
26 Tissot, F. L. H., Dauphas, N. & Grove, T. L. Distinct 238U/235U ratios and REE patterns in plutonic and volcanic angrites: Geochronologic implications and evidence for U isotope fractionation during magmatic processes. *Geochimica et Cosmochimica Acta* **213**, 593-617, doi:https://doi.org/10.1016/j.gca.2017.06.045 (2017).
27 Connelly, J. N., Bizzarro, M., Thrane, K. & Baker, J. A. The Pb–Pb age of Angrite SAH99555 revisited. *Geochimica et Cosmochimica Acta* **72**, 4813-4824, doi:https://doi.org/10.1016/j.gca.2008.06.007 (2008).
28 Richter, S. *et al.* New average values for the n(238U)/n(235U) isotope ratios of natural uranium standards. *International Journal of Mass Spectrometry* **295**, 94-97, doi:https://doi.org/10.1016/j.ijms.2010.06.004 (2010).
29 Tissot, F. L. H. & Dauphas, N. Uranium isotopic compositions of the crust and ocean: Age corrections, U budget and global extent of modern anoxia. *Geochimica et Cosmochimica Acta* **167**, 113-143, doi:https://doi.org/10.1016/j.gca.2015.06.034 (2015).
30 Gattacceca, J. *et al.* The Meteoritical Bulletin, No. 109. *Meteoritics & Planetary Science* **56**, 1626-1630 (2021).
