# Supplementary material for: Igneous meteorites suggest Aluminium-26 heterogeneity in the early Solar Nebula
Source: Nat Commun. 2023 Aug 29;14:4940. doi: 10.1038/s41467-023-40026-1 (PMC10465487; doi:10.1038/s41467-023-40026-1)
Supplement: Supplementary file 3 — Description of Additional Supplementary Files [file 41467_2023_40026_MOESM3_ESM.docx]

Description of Additional Supplementary Files

Supplementary Data 1 – U-Pb data for Erg Chech 002 from three analytcal sessions.
